# Comparing four laboratory three-parent techniques to construct human aged non-surrounded nucleolus germinal vesicle oocytes: A case-control study

**DOI:** 10.18502/ijrm.v13i6.7284

**Published:** 2020-06-30

**Authors:** Sara Darbandi, Mahsa Darbandi, Ashok Agarwal, Hamid Reza Khorram khorshid, Mohammad Reza Sadeghi, Sandro C. Esteves, Pallav Sengupta, Sulagna Dutta, Zohreh Fathi, Hojjat Zeraati, Mohammad Mehdi Akhondi

**Affiliations:** ^1^Reproductive Biotechnology Research Center, Avicenna Research Institute, ACECR, Tehran, Iran.; ^2^American Center for Reproductive Medicine, Cleveland Clinic, Cleveland, Ohio, USA.; ^3^Genetics Research Center, University of Social Welfare and Rehabilitation Sciences, Tehran, Iran.; ^4^Androfert, Andrology and Human Reproduction Clinic, Campinas, Brazil.; ^5^Department of Physiology, Faculty of Medicine, Mahsa University, Selangor, Malaysia.; ^6^Department of Epidemiology and Biostatistics, School of Public Health, Tehran University of Medical Sciences, Tehran, Iran.

**Keywords:** Assisted reproductive techniques, In vitro oocyte maturation techniques, Nuclear transfer techniques, Oocytes, Oocyte donation.

## Abstract

**Background:**

The three-parent assisted reproductive technique may increase oocyte competence.

**Objective:**

In this case-control study, the suitability of germinal vesicle transfer (GVT), synchronous ooplasmic transfer (sOT), asynchronous ooplasmic transfer using cryopreserved MII oocyte (caOT), and asynchronous ooplasmic transfer using waste MII oocyte (waOT) for maturation of the human-aged non-surrounded nucleolus germinal vesicle-stage (NSN-GV) oocyte were investigated.

**Materials and Methods:**

NSN-GV oocytes were subjected to four methods: group A (GVT), B (sOT), C (caOT) D (waOT), and E (Control). The fusion rates, MI, MII, ICSI observations and cleavage at 2-cell, 4-cell, and 8-cell stages were compared in the groups.

**Results:**

In GVT, none of the oocytes fused. In sOT, all oocytes fused, 20 achieved the MI, 14 progressed to MII, 8 fertilized, 6 cleaved and 5, 4, and 3 achieved the 2-cells, 4-cells and 8-cells, respectively. In caOT, all oocytes fused and achieved the MI, 8 progressed to MII and fertilized, 6 cleaved and 6, 5, and 5 achieved the 2-cells, 4-cells, and 8-cells respectively. In waOT, all oocytes fused, 5 and 3 progressed to MI and MII, respectively, but only one fertilized, cleaved and reached a 4-cells stage. In group E, 6 and 2 oocytes progressed to MI and MII, respectively, and only one fertilized but arrested at the zygote stage. caOT had the highest survival rate when compared to sOT (p = 0.04), waOT (p = 0.002), and control (p = 0.001).

**Conclusion:**

The caOT method was beneficial over sOT, waOT, and GVT in supplementing the developmental capacity of human-aged NSN-GV oocytes.

## 1. Introduction

In vitro maturation (IVM) of human oocytes has contributed to several births withstanding variations in patient disorders (such as premature ovarian failure and polycystic ovary syndrome), oocyte types (such as germinal vesicle [GV] and MI), and culture media. However, IVM success rates in terms of live birth remain low as compared to in vivo maturation (1-3). The insufficiency of suitable culture media and deficiencies in the oocytes due to variations in their developmental capacity might explain the meager effectiveness of IVM in assisted reproduction (3, 4). Moreover, both the in vivo and in vitro embryonic developmental success depends on the initial oocyte status and, especially, the quality of the GV oocyte (5).

The GV oocyte pool comprises two major types, namely, the “not-surrounded nucleolus” (NSN) and the “surrounded nucleolus” (SN) (6). The SN oocytes account for nearly 70-80% of GV oocyte pool and are likely the ones that naturally ovulate and endure the complete embryonic development after both in vivo and IVM and fertilization (6). The NSN oocytes account for the remaining 20-30%, which have stopped growing at the 2- or 4-cell stage after fertilization, as observed in IVM and in vitro fertilization studies (7-9).

The oocyte quality is a necessary factor to drive embryonic development via renewal, maturation, fertilization, and implantation (5). Oocyte quality includes numerous functional events like cellular organizations, metabolism, protein translation, DNA transcription, and several other factors that need to be considered (5). Nevertheless, women at advanced reproductive age often have a poor ovarian reserve. As a result, the oocyte cohort obtained after ovarian stimulation is usually limited and of poor quality overall, irrespective of the regimen used for ovarian stimulation (10, 11). The development of methods for the maturation of NSN-GV oocytes is advantageous as they might increase the number of developmentally competent oocytes for in vitro fertilization.

Ten methods have been used to increase the ooplasmic maturation, including nuclear transfer, cytoplasmic (ooplasmic) transfer, allotropic rescue, import of normal RNAs to mitochondria, mitochondrial injection, injection of mtDNA, usage of mitochondrial transcription factor A (TFAM), usage of the inner mitochondrial membrane potential (MMP) and free Ca2+ levels, dietary supplementation with cofactors and vitamins, and gonadotropin injections (12). The nuclear transfer may be performed in three oocyte developmental stages containing the GV, MII, and pronuclear (PN) (12). The two types of OT techniques are synchronous ooplasmic transfer (sOT) and asynchronous ooplasmic transfer (aOT) (4). In this project, germinal vesicle transfer (GVT), sOT, aOT using cryopreserved MII oocytes (caOT), and aOT using waste MII oocytes (waOT) have been used to improve the human aged NSN-GV oocytes' quality.

Our group has been focused on finding ways to improve the growth, viability, and overall quality of immature human aged NSN-oocytes. Our aim has been to find remedies to make such oocytes fit for use in ART thereby allowing the formation of a viable and healthy embryo. In this study, we compare for the first time the effectiveness of four three-parent techniques for the maturation of aged GV oocytes.

## 2. Materials and Methods

### Source of human oocytes

In this case-control study, the oocytes were provided through female partners of infertile couples who underwent ART treatment at the Avicenna infertility clinic affiliated with Avicenna Research Institute, Tehran, Iran, from September 2016 to September 2017. None of the volunteers had any history of chronic diseases (diabetes, cancer, etc.) or any genital inflammation, chromosomal aberrations, and endocrine abnormality.

The following four types of donated oocytes were utilized in this study from women undergoing intracytoplasmic sperm injection (ICSI) after standardized controlled ovarian stimulation: (1) discarded-aged NSN-GV oocytes obtained from participants aged 38 yr and older, (2) discarded young SN-GV oocyte from participants aged 34 yr and younger, (3) cryopreserved good-quality young MII oocytes from participants aged 34 yr and younger, and (4) waste low-quality young MII oocyte from participants aged 34 yr and younger.

### Controlled ovarian stimulation, oocyte handling 

The cumulus cell-oocyte complexes (COCs) were retrieved from women who underwent controlled ovarian hyperstimulation with recombinant human FSH (r-hFSH, Merck Fertility, Germany) after pituitary suppression with gonadotropin-releasing hormone (GnRH, Merck Fertility, Germany) agonist. The r-hFSH dose was 200 IU/day for the first four days and was adjusted on the fifth day accordingly, if needed, based on the volume and number of ovarian follicles and the serum levels of estradiol (E2). When at least three follicles became 18 mm, the COCs were retrieved transvaginally 36 hr after hCG trigger (5,000 or 10,000 IU) (Merck Fertility, Germany). The cumulus-corona cells (CCs) of COCs were removed chemically by a short-term exposure (the 40 sec) of them to medium (G-MOPS${}^{\mathrm{TM}}$PLUS, Vitrolife, Sweden) with 80 IU/mL hyaluronidase (Sigma, USA) and repetitious pipetting. Subsequently, all oocytes were considered for development with the inverted microscope at 200× or 400× magnification (13, 14).

### Oocyte cryopreservation procedures

The cryopreservation protocol involved an oocyte vitrification method. Only MII oocytes were vitrified at about 20°C based on a protocol (15). The oocytes were placed in the vitrification solution (VS) for 90 sec. According to this, oocytes were equilibrated for 1 min in 20 μl medium (G-MOPS${}^{\mathrm{TM}}$PLUS, Vitrolife, Sweden). Following oocytes were placed in 20 μl equilibration solution (ES) (Vitrolife, Sweden) with 7.5% (v/v) dimethyl sulphoxide (DMSO) (Sigma, USA) and 7.5% (v/v) ethylene glycol (EG) (Sigma, USA) for 2 min, and after that into another 20 μl ES for 2 min. The oocytes were exposed to the 20 μl ES for 5 min and then into three 20 μl drops of VS composed of 0.5 M sucrose (Sigma, USA), 15% (v/v) DMSO, and 15% (v/v) EG and were then loaded in plastic straws (Cryotpo, Kitazato, Japan) and put into cooled protective straws in liquid nitrogen for cryopreservation. Two or three oocytes were vitrified on one straw and the straws were moved into liquid nitrogen tanks and kept until thawing.

To thaw, the straws were taken from liquid nitrogen tanks and placed in 1-ml 1.0 M sucrose (37°C) for 1 min. After that, oocytes were moved to 1-ml 0.5 M sucrose for 3 min and to 1 ml medium (G-MOPS${}^{\mathrm{TM}}$PLUS, Vitrolife, Sweden) for 10 min at 20°C. Finally, oocytes were washed and cultured in the medium (G-MOPS${}^{\mathrm{TM}}$PLUS, Vitrolife, Sweden) with 10% serum protein substitute (SPS, IVFonline CT, USA) for 1 hr before manipulation. After 1 hr, the oocytes were assessed. The oocytes with normal size, intact zona pellucida clear perivitelline space, and well plasma membrane were defined as “survived.”

### Experimental design 

#### Three-parent techniques experimental groups

**Figure 1:**
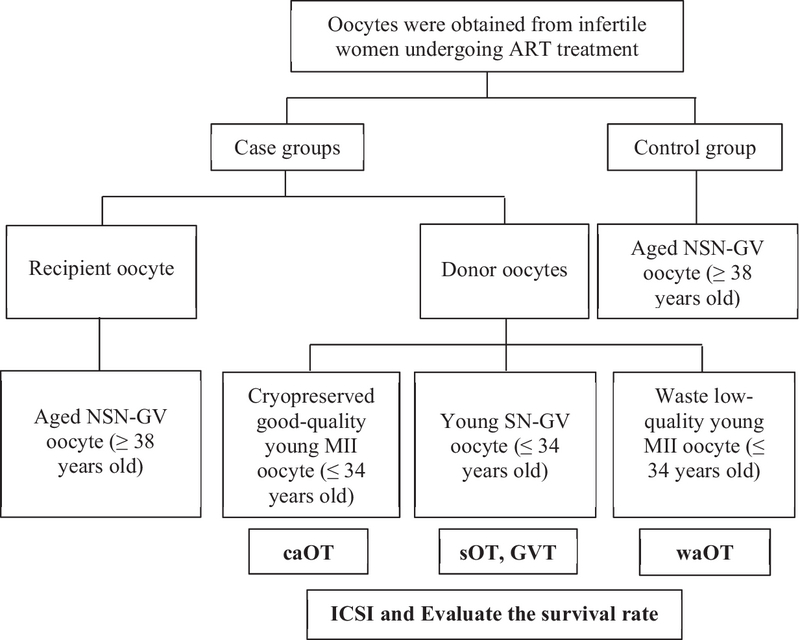
Flow diagram showing the study design.

The young fresh SN-GV oocytes, cryo-thawed young MII oocytes, and waste young fresh MII oocytes were used as an auxiliary cell for providing ooplasmic substrates for recipient fresh NSN-GV-aged oocytes. Four experimental groups were established according to the category of oocyte providing this substrate to NSN-GV oocytes as follows: Group A (GVT, n = 10), Group B (sOT, n = 26), Group C (asynchronous ooplasmic transfer using caOT, n = 12), Group D (asynchronous ooplasmic transfer using waOT, n = 12), and Group E (Control, aged NSN-GV oocytes from participants aged 38 and older, n = 12) (Figure 1 showed the study design).

The developmental stage and status of oocyte were confirmed using the inverted microscope (Diaphot, Nikon Corporation, Tokyo, Japan) at 200× magnification. Oocyte manipulation, GVT, sOT, caOT, and waOT were started within 5 hr after oocyte denudation. A total of 72 oocytes have been investigated in this study.

#### Germinal vesicle transfer (GVT)

**Figure 2:**
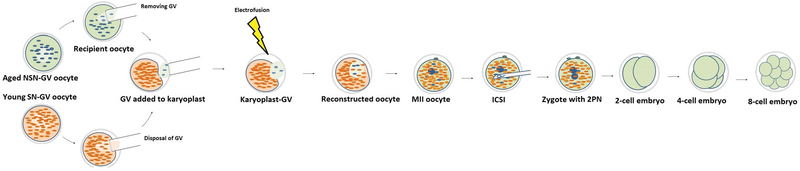
Illustration of aged NSN-GV oocytes treated with germinal vesicle transfer (GVT) method. Naked NSN-GV aged-oocytes were obtained by removal of nucleus material “directly” with a small amount of surrounding cytoplasm (karyoplast). The GV-karyoplast was inserted subzonally into an enucleated young oocyte. Membrane fusion between oocyte and karyoplast was performed by electrofusion at room temperature. All treated oocytes that reached to MII stage were subjected to sperm injection. ICSI, Intra cytoplasmic sperm injection; PN, pronuclear; NSN, not surrounded nucleolus; SN; surrounded nucleolus.

In group A, naked NSN-GV aged-oocytes were incubated in medium (G-MOPS${}^{\mathrm{TM}}$PLUS, Vitrolife, Sweden) with 7.5 µg/ml cytochalasin B (CB, Sigma, USA), 50 µg/ml 3-isobutyl-1-methylxanthine (IBMX, Sigma, USA) for 1 hr at 37°C and 6% CO${}_{2}$ (16). Precise localization of nuclear material was performed by moving oocytes in the manipulating medium (G-MOPS${}^{\mathrm{TM}}$PLUS, Vitrolife, Sweden) containing 12% PVA, 50 µg/ml IBMX, and 7.5 g/ml CB. The removal of nucleus material was carried out “directly” with a little surrounding ooplasm with the aid of a tapered manipulating glass pipette (ID: 25 µm, QG: 45° and θA: 35°) (Dain bioassay Co., Iran). The pipette was inserted through a slit made in the overlying zona pellucida using the XYClone${}^{\mathrm{TM}}$ laser system (Hamilton Thorne Biosciences, Beverly, MA, USA) and lanced the zona pellucida directly covering the GV (16). The pipette was withdrawn and the pressure inside the holding pipette (OD: 100-150 µm, ID: 30-35 and θA: 30°) was increased gently to eject the GV via the opening. The GV was injected into an enucleated young oocyte subzonally (16). Before the removal of GV, to reduce cohesion of the membranes within the pipette, the pipettes were rinsed in polyvinyl pyrrolidone 10% (PVP, Sigma, USA). Following two mineral oil droplets were used to control the fluid flow during the manipulation (17). Subsequently, the reconstructed oocytes (ROs) were rinsed three times and incubated in medium (G-IVF${}^{\mathrm{TM}}$ -Vitrolife) at 37°C for at least 30 min until electrofusion (16, 18). Membrane fusion between oocyte and GV was achieved in a microfusion chamber containing a solution of 0.3 M mannitol (Sigma, USA), 0.1 mM calcium chloride (Sigma, USA), 0.05 mg/mL human serum albumin (Sigma, USA), and 0.1 mM magnesium sulfate (Sigma, USA) at 20°C. After manual alignment, a single direct current (DC) pulse (70 V/cm DC for 20 µs) was used as a fusion pulse by the use of an ElectroCell Fusion instrument (BTX 2001, BTX, USA) (16). The GVT manipulation of aged NSN-GV oocytes is illustrated in Figure 2. Finally, the oocytes were washed and cultured (G-MOPS${}^{\mathrm{TM}}$PLUS, Vitrolife, Sweden) with 10% serum protein substitute (SPS, IVF online CT, USA) before ICSI.

#### Ooplasmic transfer (OT)

**Figure 3:**
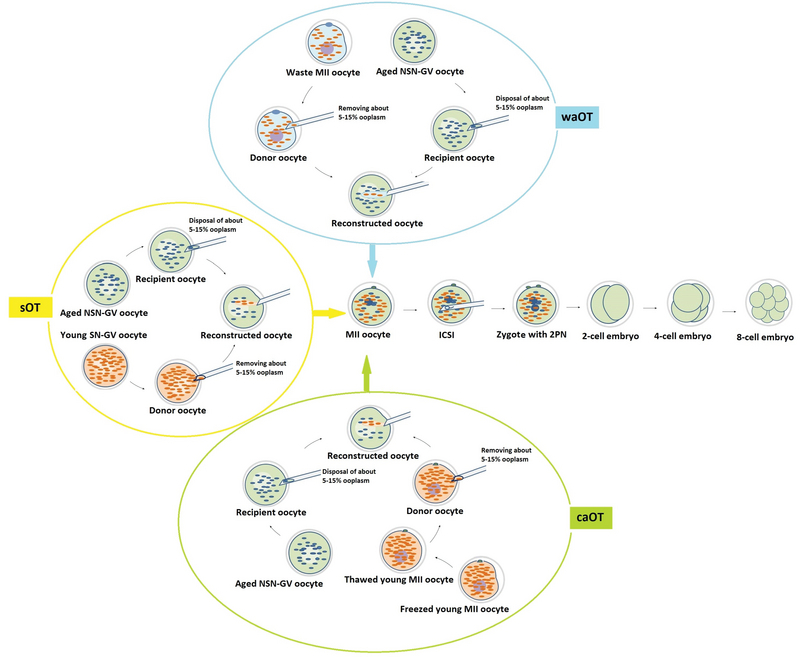
Illustration of aged NSN-GV oocytes treated with three methods of ooplasmic transfer (OT) including synchronous ooplasmic transfer (sOT), asynchronous ooplasmic transfer using cryopreserved MII oocyte (caOT), and asynchronous ooplasmic transfer using waste MII oocyte (waOT). In sOT, the ooplasm of the young SN-GV replaced that of the aged NSN-GV, while in caOT and waOT, the replacement of ooplasm was done with young MII oocytes to an aged oocyte at an NSN-GV stage of development. All treated oocytes that received to MII stage were subjected to sperm injection. ICSI, Intra cytoplasmic sperm injection; PN, pronuclear; NSN, not surrounded nucleolus; SN; surrounded nucleolus.

Figure 3 showed aged NSN-GV oocytes treated with three methods of OT including sOT, caOT, and waOT. As shown in Figure 3, in synchronous transfer (Group B), the ooplasm of the young SN-GV replaced that of the aged NSN-GV, while in asynchronous transfer (Groups C and D), the replacement of ooplasm was done with young MII oocytes (cryopreserved and wasted, respectively) to an aged oocyte at an NSN-GV stage of development (4, 12). In the OT technique, about 5-15% of ooplasm was removed from the recipient and replaced with donor ooplasm at 37°C (4, 19). Notably, oocytes received ooplasm with volume more than twice had no reprogramming potential (4, 19). This technique was performed by injecting the donor ooplasm into the recipient by a standard ICSI micropipette (4). The OT techniques were performed on the heated plate (37°C; THN-60/16 and MS 100 Controller from Linkam Scientific Instruments Ltd., London, UK) of an inverted microscope at 200× or 400× magnification (Diaphot, Nikon Corporation, Tokyo, Japan) by the Hoffman Modulation Contrast System (Modulation Optics Inc., Greenvale, NY, USA). The microscope had two coarse positioning manipulators (3D Motor-Driven Coarse control Manipulator MM-188, Narishige) and two three-dimensional hydraulic remote-control micromanipulators (Joystick Hydraulic Micromanipulator MO-188, Narishige, Japan). The oocytes were rinsed and cultured in the medium (G-MOPS${}^{\mathrm{TM}}$PLUS, Vitrolife, Sweden) with serum protein substitute 10% (SPS, IVF online CT, USA) before ICSI.

#### Semen preparation 

The fresh semen specimens were donated by the normozoospermic male spouse of couples registered in infertility clinic at the Avicenna Research Institute, Tehran, Iran. None of the subjects had any history of azoospermia, leukocytospermia, chronic diseases (diabetes, cancer, etc.), endocrine abnormalities, pelvic and genital infections, Y-chromosome microdeletion, and chromosomal aberrations. The assessment of sperm motility and the count was done based on the approvals of the World Health Organization (20). Sperm parameters were normal when total sperm motility (progressive + nonprogressive) ≥ 40%, normal sperm morphology ≥ 4%, vitality ≥ 58%, and sperm concentration was ≥ 15 million/ml (20). Semen was permitted to liquefy at 20°C for 30 min, and the density gradient centrifugation (DGC) method at 300g for 20 min with PureSpermⓇ (Nidacon, Gothenberg, Sweden) was used to separate mature sperm.

#### Intracytoplasmic sperm injections and embryo culture

All treated oocytes that reached the MII stage were subjected to sperm injection (16). Only one spermatozoon from the sperm droplet was aspirated, tail-first, into the injection pipette. The oocyte was fixed by the negative pressure of the holding pipette in place of the polar body (PB) was at 12 or 6 o'clock position. The injection pipette was hard-pressed at 3 o'clock to the zona pellucida, the oolemma, and the ooplasm below it while a spermatozoon with about 1-2 picolitres of the medium was inserted into the ooplasm.

The injection pipette was draw back calmly and the injected oocyte was leaved from the holding pipette. Then, the oocytes were washed in medium (G-1${}^{\mathrm{TM}}$, Vitrolife, Sweden), transferred into droplets of medium (G-1${}^{\mathrm{TM}}$, Vitrolife, Sweden), covered by lightweight paraffin oil (OVOIL${}^{\mathrm{TM}}$-Culture oil, Vitrolife, Sweden), and incubated (Heraeus, B5060 EK/O${}_{2}$, Van der Heyden, Brussels, Belgium; 37°C; 5% O${}_{2}$, 5% CO${}_{2}$, and 90% N${}_{2}$). About 18-20 hr after the sperm injection, the oocytes were assessed under the inverted microscope with a 200× or 400× magnification. The extrusion of second PB and formation of pronuclei were assessed too. Fertilization was normal when two clearly intact pronuclei were present. After confirmation of two pronuclei fertilization (2PN), zygotes were cultured in medium (G-1${}^{\mathrm{TM}}$, Vitrolife, Sweden) covered by paraffin oil (OVOIL${}^{\mathrm{TM}}$-Culture oil, Vitrolife, Sweden), and incubated in an incubator (Heraeus, B5060 EK/O${}_{2}$, Van der Heyden, Brussels, Belgium; 37°C; 5% O${}_{2}$, 5% CO${}_{2}$, and 90% N${}_{2}$). Cleavage of two pronuclear (2PN) oocytes was evaluated 24 hr later. The assessment of embryo development was carried out at 48 and 72 hr after the sperm injection procedure (4). The embryo quality was counted according to the homology of the blastomer's size and the number of anucleate fragments (4, 11).

#### Outcome measures 

The primary outcome measures were the rates of fusion (number of reconstructed NSN-GV aged oocytes after manipulation), MI (number of NSN-GV aged oocytes that reached MI stage), MII (number of NSN-GV aged oocytes that reached MII stage), ICSI (number of intact oocytes after ICSI), PN observation (number of pronucleus formation following ICSI), cleavage (number of oocytes that cleaved following ICSI), and percentage of embryos reaching 2-, 4-, and 8-cell stages.

### Ethical consideration

Informed consent was achieved from all volunteers and the use of human oocytes for this study was approved by the Medical Ethics Committee of Avicenna Research Institute (93/4390-93/01/01).

### Statistical analysis

The statistical package for social sciences (SPSS) software (version 22, Chicago, IL, USA) was used for all analyses. Wilcoxon was used for pairwise comparison between groups. The Kaplan-Meier and Log rank were used for evaluation of survival function in nine developmental stages including fusion, MI, MII, ICSI, PN, cleavage, 2-cell, 4-cell, and 8-cell. The “cumulative proportion surviving at end of interval", “probability density", “hazard rate", “number entering interval", “proportion terminating", and “proportion surviving" rate at all developmental stages were compared between groups. The “cumulative proportion surviving at the end of interval" related to the proportion of the cell surviving until the end of the developmental stage. The “number entering interval" showed the denominator cell number for each time interval. The “proportion terminating" represented the proportion of NSN-GV oocytes that had died. The “proportion surviving" represented the proportion of NSN-GV oocytes that had survived during the development. The “probability density" and “hazard rates" for the life table and the height of the survival curve were presented too. In all statistical analyses, a P-value ≤ 0.05 was set as significant. In all tables, four groups of B, C, D, and E were compared and group A has been removed due to lack of changes and only was descripted in the text. The sample size was based on previous studies (21). However, since aged women usually have a low ovarian reserve and most of their retrieved oocytes are used for their own treatment, we found it very hard to obtain these samples for this investigation.

## 3. Results

The descriptive and comparative statistics of different methods utilized for improving the fertilization and developmental potential of human aged NSN-GV oocytes are shown in Table I and the life table of different methods utilized for improving the fertilization and developmental potential of human aged NSN-GV oocytes are shown in Table II. In group A, none of the oocytes fused, so this group has been removed from the analysis shown in the tables. In group B, all oocytes fused and 20 achieved the MI stage. Of these, 14 progressed to MII and 8 were fertilized. The pronuclear formation was observed in six resulting zygotes, all of which cleaved and five, four, and three achieved a 2-cell, 4-cell and 8-cell stage, respectively (Tables I, II). In group C, all oocytes fused and achieved the MI stage. Of these, eight progressed to MII and showed signs of fertilization. Of the six cleaved embryos, six, five, and five achieved a 2-cell, 4-cell, and 8-cell stage, respectively (Tables I, II). In group D, all oocytes fused, five and three progressed to MI and MII, respectively, but only one fertilized. This zygote cleaved and reached a 4-cell stage embryo (Tables I, II). In group E, six and two oocytes progressed to MI and MII stages, respectively. Of these, only one fertilized but arrested at the zygote stage (Tables I, II).

**Figure 4:**
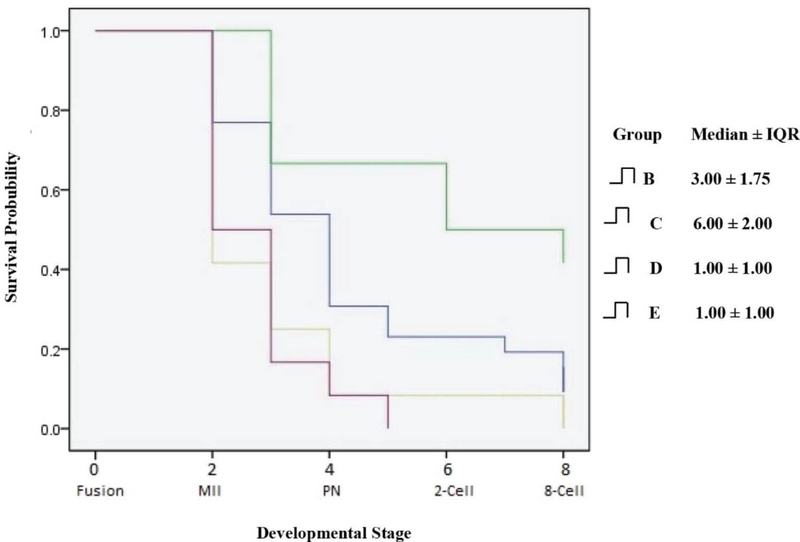
Developmental potential of human aged NSN-GV oocytes in group B, C, D, and E. Generalized Wilcoxon was used for comparisons (Chi-Square:16.53, df: 3, P-value: 0.001). Group A, germinal vesicle transfer (GVT); group B; synchronous ooplasmic transfer (sOT); group C, asynchronous ooplasmic transfer using cryopreserved MII oocyte (caOT); group D, asynchronous ooplasmic transfer using waste MII oocyte (waOT); and group E, control. NSN, not surrounded nucleolus; SN, surrounded nucleolus.

**Figure 5:**
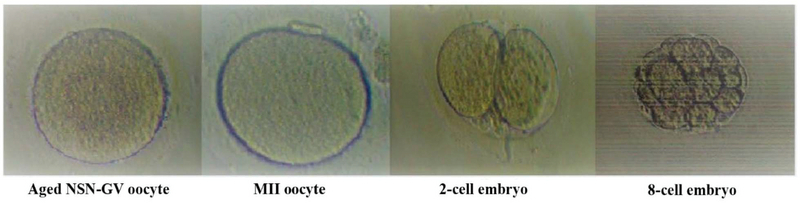
A human aged NSN-GV oocyte that achieved the 8-cell stage development observed by invert microscopy (400× magnification). In our study, cleavage zygotes were observed with all but GVT methods. However, asynchronous ooplasmic transfer using cryopreserved MII oocytes (caOT) resulted in more zygotes achieving the 2-cell, 4-cell, and 8-cell stages than the other methods.

The cumulative survival rate, probability density, hazard ratio, the number entering an interval, proportion terminating, and the surviving rate at all developmental stages were compared between groups (Table II). A human aged NSN-GV oocyte that achieved the 8-cell stage development has been illustrated in Figure 4. We found that group C had a higher survival rate than groups B (p < 0.05), D (p < 0.005), and E (p < 0.001) (Table I). Cumulative survival rate at the 8-cell stage was respectively higher in group C (0.42) and B (0.12) than the other groups (Table II). In other words, this gives 42% and 12% probability of surviving up to the 8-cell stage following caOT and sOT, respectively, in groups C and B. The Proportion Surviving represents the proportion of NSN-GV oocytes that survived during the development. Therefore, for groups C and B, the probability of surviving during 8-cell development was 1.00 (approximately 100%) (Table II). The developmental potential of human aged NSN-GV oocytes in groups B, C, D, and E are shown in Figure 5.

**Table 1:** Descriptive and comparative statistics of different methods utilized for improving the fertilization and developmental potential of human aged NSN-GV oocytes

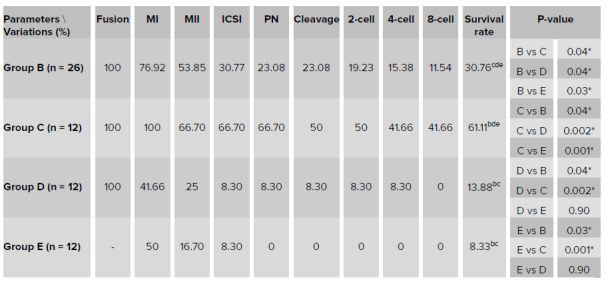
Wilcoxon was used for pairwise comparison between groups. Here, four groups of B, C, D and E were compared. Group A has been removed due to lack of changes and only has only been descripted in the text. The data were reported as a percentage. In this table, nine parameters related to the fertilization, and developmental potential of oocytes including fusion, MI, MII, ICSI, PN, cleavage, 2-cell, 4-cell, and 8-cell were compared between groups. For analyzing and comparing the data, the Kaplan-Meire and Log rank were used for the evaluation of survival function in the nine developmental stages. The P-value of comparing “survival rates” between groups have been shown in the last column of this table. *P ≤ 0.05; significant, **P ≤ 0.001; highly significant. Group A, germinal vesicle transfer (GVT); Group B, synchronous ooplasmic transfer (sOT); Group C, asynchronous ooplasmic transfer using cryopreserved MII oocyte (caOT); Group D, asynchronous ooplasmic transfer using waste MII oocyte (waOT); Group E, control group. ICSI, Intra cytoplasmic sperm injection; PN, pronuclear

**Table 2:** Life table of different methods utilized for improving the fertilization and developmental potential of human aged NSN-GV oocytes


**Group**	**Fusion**	**MI**	**MII**	**ICSI**	**PN**	**Cleavage**	**2-cell**	**4-cell**	**8-cell**
**Cumulative proportion surviving at end of interval**									
**B**	0.77	0.54	0.31	0.23	0.23	0.19	0.15	0.12	0.12
**C**	1.00	0.67	0.67	0.67	0.50	0.50	0.42	0.42	0.42
**D**	0.42	0.25	0.08	0.08	0.08	0.08	0.08	0.00	-
**E**	0.50	0.17	0.08	0.00	- -	- -	-
**Probability density**									
**B**	0.23	0.23	0.23	0.08	0.00	0.04	0.04	0.04	0.00
**C**	0.00	0.33	0.00	0.00	0.17	0.00	0.08	0.00	0.00
**D**	0.58	0.17	0.17	0.00	0.00	0.00	0.00	0.08	
**E**	0.50	0.33	0.08	0.08	- -	- -	-
**Hazard rate**									
**B**	0.26	0.35	0.55	0.29	0.00	0.18	0.22	0.29	0.00
**C**	0.00	0.40	0.00	0.00	0.29	0.00	0.18	0.00	0.00
**D**	0.82	0.50	1.00	0.00	0.00	0.00	0.00	2.00	-
**E**	0.67	1.00	0.67	2.00	- -	- -	-
**Group**	**Fusion**	**MI**	**MII**	**ICSI**	**PN**	**Cleavage**	**2-cell**	**4-cell**	**8-cell**
**Number entering interval**									
**B**	26	20	14	8	6	6	5	4	3
**C**	12	12	8	8	8	6	6	5	5
**D**	12	5	3	1	1	1	1	1	0
**E**	12	6	2	1	0	0	0	0	0
**Proportion terminating**									
**B**	0.23	0.30	0.43	0.25	0.00	0.17	0.20	0.25	0.00
**C**	0.00	0.33	0.00	0.00	0.25	0.00	0.17	0.00	0.00
**D**	0.58	0.40	0.67	0.00	0.00	0.00	0.00	1.00	-
**E**	0.50	0.67	0.50	1.00	- -	- -	-
**Proportion surviving**									
**B**	0.77	0.70	0.57	0.75	1.00	0.83	0.80	0.75	1.00
**C**	1.00	0.67	1.00	1.00	0.75	1.00	0.83	1.00	1.00
**D**	0.42	0.60	0.33	1.00	1.00	1.00	1.00	0.00	-
**E**	0.50	0.33	0.50	0.00	- -	- -	-
The Kaplan-Meire and Log-rank were used for the evaluation of survival function in nine developmental stages including Fusion, MI, MII, ICSI, PN, Cleavage, 2-cell, 4-cell, and 8-cell. Here, four groups of B, C, D, and E were compared. Group A has been removed due to the lack of changes and has only been described in the text. Group A, germinal vesicle transfer (GVT); Group B, synchronous ooplasmic transfer (sOT); Group C, asynchronous ooplasmic transfer using cryopreserved MII oocyte (caOT); Group D, asynchronous ooplasmic transfer using waste MII oocyte (waOT); Group E, control group. ICSI, Intra cytoplasmic sperm injection; PN, pronuclear; NSN, not surrounded nucleolus; SN, surrounded nucleolus. Cumulative proportion surviving at the end of interval: the proportion of the cell surviving until the end of the developmental stage; Proportion surviving: the proportion of NSN-GV oocytes which survived during development; Proportion terminating: the proportion of NSN-GV oocytes which died; Number entering interval: the denominator cell number for each time interval									

## 4. Discussion 

To the best of our knowledge, this is the first study to compare the effectiveness of methods aiming at improving the reproductive potential of human aged NSN-GV oocytes. We evaluated four three-parent techniques and found that the caOT technique seems to be advantageous over sOT, waOT, and GVT concerning the developmental capacity of human-aged NSN-GV oocytes. Notwithstanding these promising results, our study has several limitations as the number of NSN-GV oocytes utilized was small due to the inherent difficulties to obtain human oocytes for research.

GVT has been the most studied method to improve oocyte quality. It involves the removal of nuclear chromosomal content from aged oocytes and transplantation into enucleated healthy oocytes (12, 16, 22). In this study, we were not successful in achieving embryo development in any aged NSN-GV RO, owing to the problems in gathering the nuclear structure even after using medium supplemented with IBMX. Studies in other mammal species reported variable success with GVT (16). The discrepancy between our results and those of others might be attributable to the use of different oocyte source. Previous studies lack information concerning the type of GV oocyte utilized - SN or NSN. We speculate that GVT was too invasive for use with NSN-GV oocytes. It has been shown that from mitochondrial dysfunction of aging, oocytes might cause peroxidative damage to nuclear DNA or cell membranes, ultimately resulting in cellular function decay (4, 12). Furthermore, studies reported that some oocytes that had matured in vitro underwent apoptosis (4, 12). It seems that these mechanisms can explain the failure of GVT in aged NSN-GV oocyte.

By contrast, our results suggest that the three other methods might be potentially useful to improve oocyte quality of aged NSH-GV oocytes. As compared with group A (GVT), the rates of oocyte fusion were 100% in groups B (sOT), C (caOT), and D (waOT).

After oocyte fusion, GV oocytes have to progress to the next developmental stages. The extrusion of the first and second polar bodies is used as markers of nuclear maturation and meiosis completion (23, 24). In this study, the mean percentage of oocytes achieving the MI stage was 0% in GVT compared with 76.9% in sOT, 100.0% in caOT, 41.7% in wOT, and 50.0% in the control group (Group E). The ratio of oocytes reaching the MI stage was significantly higher in the sOT and caOT than in the waOT and control oocytes. Likewise, the percentage of oocytes arrived at MII stage was significantly higher in sOT, caOT, and wOT than in the GVT and control groups.

The extrusion of the second PB and formation of pronuclei occur approximately 18-20 hr and 20-24 hr after sperm injections, respectively (12). Our results indicate that the likelihoods of NSN aged-GV-treated oocytes to be fertilized are very low (< 10%) with GVT or wOT methods. By contrast, fertilization rates were highest with caOT (∼66%) followed by sOT (∼30%), and both methods had higher 2PN fertilization rates than controls (∼8%). Notably, in this study, we showed that the 2PN fertilization rate observed with the caOT method was similar than that of achieved after sperm injection of mature oocytes in ART programs. In our study, cleavage zygotes were observed with all but GVT methods. However, asynchronous ooplasmic transfer using cryopreserved MII oocytes [caOT] resulted in more zygotes achieving the 2-cell, 4-cell, and 8-cell stages than the other methods.

Our results indicate that cytoplasmic transfer into human aged NSN-GV oocytes might improve preimplantation development. Considering all maturation and development stages, caOT was the most efficacious method to improve the fertilization and developmental capacity of aged NSN-GV. This approach might increase the reproductive success of aged women with infertility that usually has a limited and poor-quality oocyte cohort available for fertilization.

Although some studies assessed the effects of NT or OT techniques on oocyte maturation (4, 12, 16), to our knowledge, no study has evaluated the effects of these techniques on the quality of aged NSN-GV oocytes. Studies showed that NT or OT are useful to improve ooplasmic maturation in mammalian oocytes, but our result showed that the caOT method was beneficial over sOT, waOT, and GVT in supplementing the developmental capacity of human aged NSN-GV oocytes. It is reported that children born using the OT may exhibit mitochondrial heteroplasmy (12, 25, 26). However, the levels of heteroplasmy were not assessed in this study and this important aspect should be investigated in the 8-cell stage in future studies. The recipient oocyte also has other molecules and organelles that may induce genomics, transcriptomics, proteomics, metabolomics, and epigenomics alterations (12, 27). Along the same lines, the assessment of blastocyst development and embryo genetic and epigenetic status of resulting embryos were not carried out and should be investigated further.

The techniques for improving oocyte quality of mammalian oocytes are evolving. However, there are several gaps in the knowledge that need to be investigated in future research. Besides embryo genetic competence, it is important to investigate the well-being of the offspring generated. There is a risk that genetic and epigenetics modifications are incurred during the application of these methods. RNAs, proteins, mitochondria, associated structures, and energetics substrates are transferred from donor to recipient oocytes (12, 28), and children born using these parent techniques were shown to exhibited mitochondrial heteroplasmy (12). The complex interactions between nuclear DNA and mtDNA as well as with other molecules might cause epigenetic alterations of the nuclear genome which requires close monitoring (28).

## 5. Conclusion 

The asynchronous ooplasmic transfer using cryopreserved MII oocytes technique (caOT) seems to be advantageous over sOT, waOT, and GVT in the developmental capacity of human-aged NSN-GV oocytes. caOT holds promise concerning its possible role in augmenting the developmental capacity of human aged NSN-GV in terms of fertilization and cleavage, thus warranting further investigation.

##  Conflict of Interest

All authors declare no financial or commercial competing interest.
